# Oral immunization of mice with a probiotic *Lactobacillus casei* constitutively expressing the α-toxoid induces protective immunity against *Clostridium perfringens* α-toxin

**DOI:** 10.1080/21505594.2019.1582975

**Published:** 2019-02-26

**Authors:** Xuwen Gao, Yingying Ma, Zhuo Wang, Jing Bai, Shuo Jia, Baohua Feng, Yanping Jiang, Wen Cui, Lijie Tang, Yijing Li, Li Wang, Yigang Xu

**Affiliations:** a College of Veterinary Medicine, Northeast Agricultural University, Harbin, P.R. China; b China Ministry of Agriculture Key Laboratory of Animal Pathogen Biology, Northeastern Science Inspection Station, Harbin, P.R. China; c Heilongjiang Key Laboratory for Animal Disease Control and Pharmaceutical Development, Northeast Agricultural University, Harbin, P.R. China

**Keywords:** *Clostridium perfringens*, α-toxoid, genetically engineered *Lactobacillus casei*, oral immunization

## Abstract

*Clostridium perfringens* α-toxin is one of the major virulence factors during *C. perfringens* infection, causing hemolysis of erythrocytes in various species. Here, genetically engineered *Lactobacillus casei* (pPG-α/*L. casei* 393) constitutively expressing the toxoid of *C. perfringens* α-toxin was generated and its immunogenicity in mice for induction of protective immunity against the α-toxin was evaluated via oral immunization. The α-toxoid was constitutively expressed by pPG-α/*L. casei* 393 without a specific inducer, as confirmed by western blotting, laser confocal microscopy, and flow cytometry. In an experiment on BALB/c mice to evaluate the oral immunogenicity of pPG-α/*L. casei* 393, significant levels of a specific secretory IgA (sIgA) antibody in the intestinal mucus and feces and an IgG antibody in the serum of the probiotic vaccine group were detected after booster immunization (*p *< 0.05) as compared with the pPG/*L. casei* 393 and PBS control groups. These antibodies effectively neutralized *C. perfringens* natural α-toxin. Moreover, significantly higher levels of cytokines IL-2, IL-4, IL-10, IL-12, IL-17, and interferon (IFN) γ in the serum and increased proliferation of spleen lymphocytes obtained from mice orally immunized with pPG-α/*L. casei* 393 were detected. With a commercial *C. perfringens* type A inactivated vaccine as a control, immune protection provided by the probiotic vaccine against *C. perfringens* α-toxin was evaluated, and 90% and 80% protection rates were observed, respectively. Therefore, strain pPG-α/*L. casei* 393 effectively elicited mucosal, humoral, and cellular immunity, suggesting that pPG-α/*L. casei* 393 is a promising candidate for development of a vaccine against *C. perfringens* α-toxin.

## Introduction

*Clostridium perfringens*, a gram-positive, spore-forming anaerobic bacterium, is ubiquitous in the environment, e.g. in soil, feces, sewage, and human and animal intestinal tracts [1,2]. It is also one of the most common pathogens that can cause histotoxic and enterotoxic diseases in humans and animals (mainly cattle and sheep), e.g. food poisoning in humans and necrotic enteritis, intestinal toxemia, and traumatic gas gangrene in animals, as a result of the ability to produce potent extracellular toxins [3,4]. The *C. perfringens* toxinotyping scheme has been helpful for diagnosing *C. perfringens* infections in humans and animals. On the basis of the traditional scheme of a combination of four typing toxins (α-toxin, β-toxin, ϵ-toxin, and ι-toxin), *C. perfringens* strains are classified into five toxinotypes: A to E [5]. Recently, authors of an updated study proposed that *C. perfringens* strains be classified into seven toxinotypes: A to G [6]. Generally, most diseases caused by *C. perfringens* in sheep, cattle, goats, and other animal species are called enterotoxemias.

As a typical inhabitant of the intestinal tract of many animal species, *C. perfringens* may proliferate to large numbers when the intestinal environment is altered by sudden changes in diet or other factors. As a result, potent toxins are produced and absorbed into the systemic circulation or act locally, having devastating effects on the host. Among these toxins, *C. perfringens* α-toxin is one of the major virulence factors, has both enzymatic and toxin properties [7], and plays a crucial role in the pathogenesis of relevant diseases [8,9]. Histopathologically, all intestinal disorders are characterized by damage to the tips of villi or by epithelial cell detachment, congestion of the capillaries, mucosal edema, and necrosis. In most cases, hemorrhage and mucosal inflammation with an influx of inflammatory cells are commonly reported [10,11]. Some studies have revealed that histidine residues at positions 11, 68, 126, 136, and 148 of *C. perfringens* α-toxin are critical for its biological activities. When these histidines are replaced by other amino acid residues, such as glycine, the hemolytic activity and lethality of the α-toxin are significantly reduced or even eliminated. Nonetheless, its antigenicity can be retained [12–14], pointing to a promising strategy for the development of a subunit vaccine against *C. perfringens* α-toxin [15,16].

Currently, in-feed antibiotics, such as virginiamycin and tylosin, are commonly used to control *C. perfringens* infections in livestock and poultry. Nevertheless, antibiotics can have many negative effects on the environment and human health. According to the characteristics of *C. perfringens* intestinal infections and intestinal absorption of *C. perfringens* enterotoxin, an effective oral vaccine that can induce specific secretory IgA (sIgA)-based mucosal and IgG-based humoral immunity against a *C. perfringens* α-toxin challenge is important for clinical practice. Lactic acid bacteria (LAB), a type of facultative anaerobic gram-positive bacteria, are widely distributed in the digestive tract, respiratory tract, and genitourinary system of humans and animals [17] and plays an important part in probiotic effects on the host, e.g. regulation of the microecology balance. Moreover, LAB and their metabolites perform the functions of nutrition and host immunity regulation [18,19]. Furthermore, genetically engineered LAB can be used to express functional proteins of pharmaceutical significance, in particular oral vaccines; this property makes such LAB attractive candidates for antigen delivery carriers for the development of mucosal vaccines [20,21]. LAB as vaccine vectors have the following attractive advantages: safety, noninvasive administration (usually oral or intranasal), good acceptance and stability of genetic modifications, and relatively low cost [22,23]. Furthermore, cell wall–associated or secreted factors from LAB strains can effectively enhance innate immune responses and epithelial barrier function, modulate the intestinal microenvironment, regulate immune-cell behavior, and elicit a cytokine release [23].

In this study, *Lactobacillus casei*, a potential antigen delivery vehicle for the development of oral vaccines [24], was employed to construct a genetically engineered probiotic vaccine delivering the toxoid of *C. perfringens* α-toxin. Immunogenicity of this vaccine in mice for induction of protective immunity against *C. perfringens* α-toxin was evaluated via oral immunization.

## Materials and methods

### Bacterial strains and plasmids

*C. perfringens* toxinotype A (C57-1) was obtained from the China Institute of Veterinary Drug Control (Beijing, China) and was grown anaerobically at 37°C in Schaedler Anaerobe Broth (Oxoid Limited, UK). *Escherichia coli* strains JM109 and TG1 and engineered strain pMD19-T-α/JM109 that carries the gene encoding the α-toxoid (in which histidine-68 in the α-toxin was mutated to a glycine residue) and strain pPG-T7g10/TG1 carrying constitutive expression plasmid pPG-T7g10 constructed by our laboratory were cultured overnight in Luria–Bertani (LB) broth at 37°C. *Lactobacillus casei* ATCC 393 kept in our laboratory was grown anaerobically in de Man, Rogosa, and Sharpe (MRS) broth (Sigma, USA) at 37°C without shaking. The details of all plasmids used in this study are listed in Table 1.

**Table 1.** Details of plasmids used in this study.

Plasmids	Relevant Characteristics	Description/Reference
pMD19-T	A cloning vector; ampicillin resistance, Amp^r^	Takara, China
pMD19-T-α	Recombinant pMD19-T containing a gene encoding the toxoid of *C. perfringens* α-toxin; Amp^r^	In this work
pET-32a	A vector for prokaryotic expression; Amp^r^	Takara, China
pET-32a-α	Recombinant pET-32a containing a gene encoding the toxoid of *C. perfringens* α-toxin for α-toxoid expression to develop monoclonal antibodies; Amp^r^	In this work
pPG-T7g10	A constitutive expression plasmid consisting of the HCE promoter, PgsA anchor, rrnBT1T2 terminator, and repA and repC replicon; chloramphenicol resistance (Cm^r^) gene	[24]
pPG-α	Recombinant pPG-T7g10 containing the gene encoding toxoid of *C. perfringens* α-toxin for constructing the genetically engineered *Lactobacillus casei*; Cm^r^	In this work

### *Construction of a recombinant expression system in* L. casei

**Figure 1.**
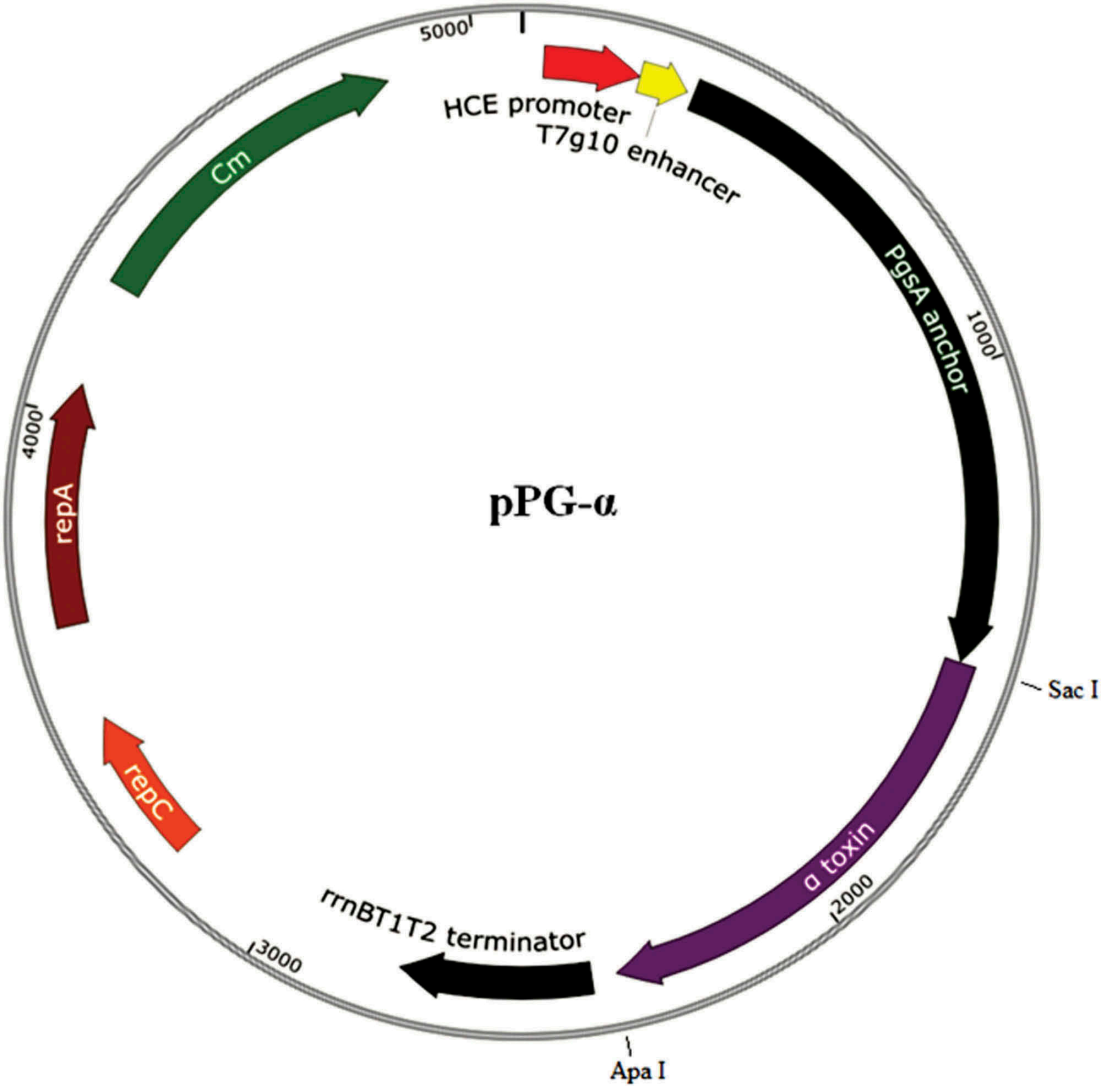
Recombinant plasmid pPG-α (containing the gene encoding the toxoid of *C. perfringens* α-toxin) constructed in this study. The gene fragment encoding the α-toxoid was obtained from plasmid pMD19-T-α constructed in our lab with *Sac* I and *Apa* I double-enzyme digestion and was inserted at the corresponding sites of pPG-T7g10, giving rise to pPG-α.

For the construction of recombinant expression plasmid pPG-α, the ampicillin (Amp)-resistant pMD19-T-α/JM109 strain and chloramphenicol (Cm)-resistant pPG-T7g10/TG1 strain were cultured overnight in LB broth at 37°C. Following the extraction of plasmids pMD19-T-α and pPG-T7g10 with the Plasmid DNA Extraction Kit (Qiagen, China) and double-enzyme digestion with *Sac* I and *Apa* I (New England Biolabs, China), the gene fragment encoding the α-toxoid of *C. perfringens* was subcloned into constitutive expression vector pPG-T7g10 constructed in our laboratory, giving rise to recombinant plasmid pPG-α (Figure 1). For preparation of *L. casei* competent cells, a single colony of *L. casei* 393 was picked from the MRS agar plate and incubated in 5 mL of antibiotic-free MRS broth at 37°C for 16 h. Next, it was transferred into 100 mL of antibiotic-free MRS broth to culture until optical density at 600 nm (OD_600_) of approximately 0.5 and was centrifuged at 3000 × *g* for 10 min at 4°C. After that, the cell pellets were washed thrice with precooled EPWB (0.6 mmol/L NaH_2_PO_4_, 0.1 mmol/L MgCl_2_) and twice with precooled EPB (EPWB supplemented with 0.3 M sucrose). The cell pellets were resuspended in EPB and stored at – 80°C until use. For construction of engineered *L. casei* bearing the pPG-α plasmid, electroporation was carried out as previously described [25], and engineered strains were identified by screening of strains on Cm-supplemented MRS-agar medium and were checked by extraction of recombinant plasmid DNA, following by restriction site analysis and sequencing.

### Protein expression analysis

The engineered *L. casei* strain carrying plasmid pPG-α (pPG-α/*L. casei* 393) was cultured in MRS broth without a specific inducer at 37°C for 12 h. After that, the bacterial cultures were centrifuged at 12,000 × *g* for 5 min, and the cells were lysed with 2× SDS buffer followed by boiling for 10 min and centrifugation at 12,000 × *g* for 10 min. The supernatant was subjected to sodium dodecyl sulfate-polyacrylamide gel electrophoresis (SDS-PAGE) in a 10% gel followed by western blot analysis using a mouse anti–α-toxoid monoclonal antibody diluted at 1:500 as the primary antibody (the monoclonal antibody was prepared by means of the α-toxoid protein expressed from the pET-32a-α/BL21 plasmid constructed in our lab) and a horseradish peroxidase (HRP)-conjugated goat anti-mouse IgG antibody (Sigma, USA) diluted 1:2000 as the secondary antibody. Next, the immunoblot was visualized with the chemiluminescent substrate reagent (Pierce, USA). In addition, to identify the α-toxoid protein expressed on the cell surface of pPG-α/*L. casei* 393, laser confocal microscopy and flow cytometry were performed. In brief, 1 mL of pPG-α/*L. casei* 393 (OD_600_ ≈ 1) was centrifuged at 5000 × *g* for 5 min, and the cells were washed thrice with sterile PBS. Then, 500 μL of a mouse anti–α-toxoid monoclonal antibody (diluted at 1:100) was added to the cells, gently mixed, and incubated at 37°C for 30 min. After centrifugation and washing with sterile PBS, 500 μL of a fluorescein isothiocyanate (FITC)-labeled goat anti-mouse IgG antibody (diluted at 1:200; Sigma, USA) was added, mixed, and incubated at 37°C for 60 min in the dark. After a wash and centrifugation, the cell pellets were resuspended in sterile PBS and used for laser confocal microscopy examination and flow-cytometric analysis.

### Oral immunization

All applicable international (OIE Terrestrial animal health code) and national guidelines (CNAS-CL06:2018) for the care and use of laboratory animals were followed. Approval was obtained from the Institutional Committee of Northeast Agricultural University for the animal experiments. In this study, to evaluate the immunogenicity of engineered strain pPG-α/*L. casei* 393 serving as an oral vaccine, 5-week-old specific pathogen-free (SPF) BALB/c mice obtained from Liaoning Changsheng Biotechnology Co., Ltd. (Liaoning, China) were chosen as an animal model and were kept under SPF conditions with free access to a standard diet and water. Before oral immunization, engineered strain pPG-α/*L. casei* 393 was cultured in MRS broth for 16 h without shaking followed by washing with PBS and was resuspended in PBS at a concentration of 10^10^ CFU/mL. The probiotic vaccine group (41 mice) was immunized with 200 μL of pPG-α/*L. casei* 393 per mouse [26]. The control group (41 mice) received an equivalent dose of pPG/*L. casei* 393, and mice (n = 41) in the mock control group received 200 μL of PBS. In parallel, mice (n = 41) were injected intramuscularly with a commercial inactivated *C. perfringens* type A vaccine obtained from China Animal Husbandry Industry Co., Ltd. (Beijing, China) and served as the vaccine control group. All mice were immunized via oral administration, and the immunization protocol was carried out on three consecutive days: days 1, 2, and 3. Booster immunization was administered on days 14, 15, and 16, and a second booster was given on days 28, 29, and 30 (as shown in Figure 3(a)) as previously described [25].

### Enzyme-linked immunosorbent assays (ELISAs)

On days 0, 7, 14, 21, 28, 35, and 42 after immunization, serum, feces, and intestinal-mucus samples were collected from three mice selected randomly in each group, and the immune samples were prepared as described elsewhere [26,27]. In brief, the serum samples were collected and stored at – 80°C until analysis; approximately 0.2 g of fecal pellets was resuspended in 500 μL of PBS supplemented with 1 mmol/L phenylmethylsulfonyl fluoride (Sigma, USA) and 1% bovine serum albumin (Sigma, USA), and were incubated overnight at 4°C followed by centrifugation. The supernatants were stored at – 80°C until analysis; the intestinal mucus samples were gently scraped from the excised intestinal tissue and were placed into 500 μL of HEPES buffer (Thermo Fisher Scientific, USA), and the suspensions were mixed well by shaking at 4°C. The supernatants were collected by centrifugation and stored at – 80°C until assays. For the ELISA, 96-well polystyrene microtiter plates were coated overnight at 4°C with the purified α-toxoid protein expressed by pET-32a-α/BL21 and were washed thrice with PBS containing 0.05% Tween 20 and saturated with 10% bovine serum albumin (BSA) at 37°C for 2 h. After that, the collected immune samples (sera diluted at 1:50; supernatants of feces and intestinal mucus diluted at 1:10) as primary antibodies were added and incubated at 37°C for 1 h, followed by washing thrice with PBS supplemented with 0.05% Tween 20. Next, an HRP-conjugated goat anti-mouse IgA antibody (Sigma, USA) or IgG antibody (Sigma, USA) diluted at 1:2000 was used as a secondary antibody to detect the bound antibodies. After that, o-phenylenediamine dihydrochloride (Sigma, USA) was employed as a substrate to develop the color, and absorbance at OD_490_ was measured. Moreover, the cytokine levels in serum samples collected on day 35 post-immunization were determined by means of an ELISA Kit (Biosource, USA), including interleukin 2 (IL-2), IL-4, IL-10, IL-12, IL-17, and interferon γ (IFN-γ).

### The neutralization ability of serum and intestinal mucosal antibodies toward the α-toxin

The neutralizing ability of the serum IgG antibody and of intestinal mucosal sIgA antibody toward *C. perfringens* natural α-toxin obtained from mice orally immunized with pPG-α/*L. casei* 393 was determined by means of the IgG and sIgA antibodies obtained from pPG/*L. casei* 393, with PBS groups as a control. Briefly, serum IgG and intestinal mucosal sIgA antibodies collected from the mice immunized with pPG-α/*L. casei* 393, pPG/*L. casei* 393, and PBS on day 35 after immunization were mixed with an equal volume (1 × LD_100_ [lethal dose]) of a solution of the natural α-toxin produced by *C. perfringens* toxinotype A (C57-1) and were incubated at 37°C for 1 h. Each group of 20 five-week-old SPF BALB/c mice was challenged with 300 μL of the α-toxin treated with the IgG antibody (by injection)/sIgA antibody (by oral administration with oral gavage needles) to examine survival of the mice.

### Proliferation of lymphocytes in immunized mice

On day 42 post-immunization, splenocytes were obtained from the mice in each group and were subjected to a lymphocyte proliferation assay. In brief, 100 μL of the splenocyte suspension (~5 × 10^6^ cells/mL) was incubated in a 96-well plate containing the RPMI 1640 medium supplemented with 10% of fetal calf serum at 37°C in a 5% CO_2_ incubator. After that, splenocytes were stimulated with the purified α-toxoid protein expressed by pET-32a-α/BL21 at a final concentration of 1, 25, and 50 μg/ml for 72 h. Finally, lymphocyte proliferation was assessed by a CellTiter 96® AQueous Non-Radioactive Cell Proliferation Assay (MTT) (Promega, USA), with absorbance measured at 570 nm.

### Immune protection by the probiotic vaccine

To evaluate the immune protection of mice orally vaccinated with pPG-α/*L. casei* 393, on day 35 postimmunization, mice (n = 20) in each group were challenged with 1 × LD_100_ of the natural α-toxin combined with 200 μL of *C. perfringens* type A (~10^9^ CFU/mL) by oral administration via an oral gavage needle. Animal health was monitored daily for a 10-day observation period, and the cumulative mortality of mice in each group was recorded. Next, histopathological changes in the heart, liver, spleen, kidneys, brain, and gut were examined with special attention to color and structure, the presence of congestion and hemorrhage, necrosis, and inflammatory-cell infiltration.

### Statistical analysis

Data are shown as mean ± standard error of three replicates per test in a single experiment repeated three times. Kaplan–Meier survival analysis was conducted too. Tukey’s multiple-comparison tests were performed to analyze the differences between groups. *P *< 0.05 was assumed to indicate statistical significance, and *P *< 0.01 high significance.

## Results

### Identification of protein expression

**Figure 2.**
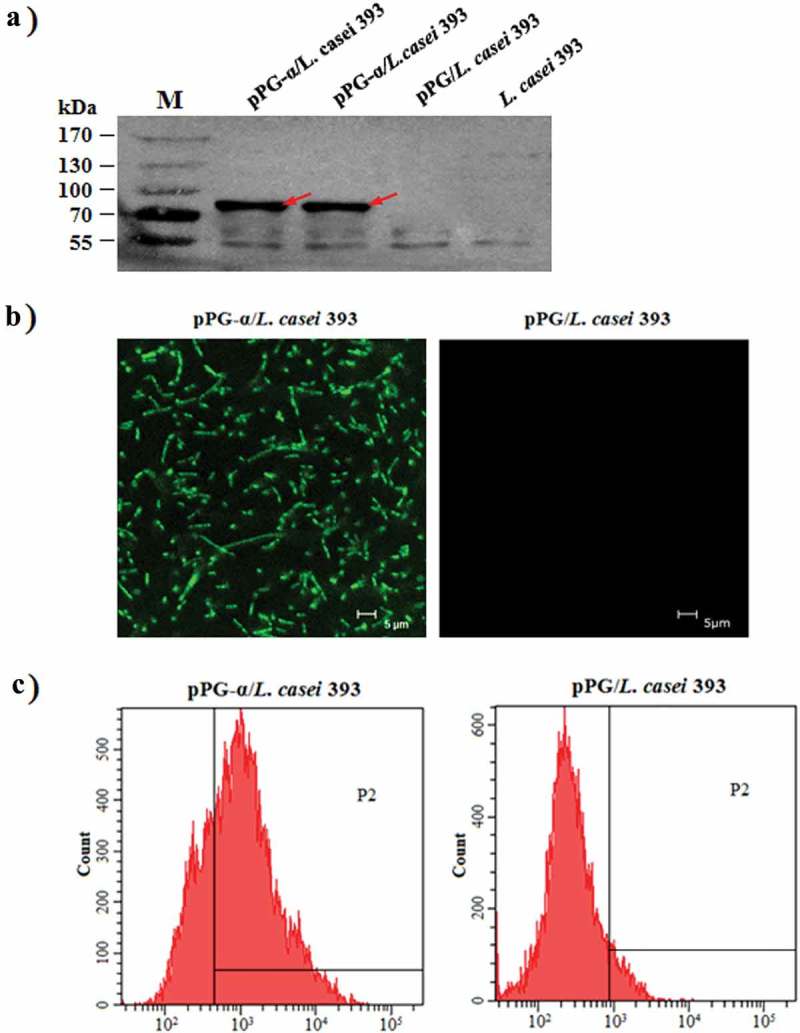
Confirmation of α-toxoid protein expression by pPG-α/*L. casei* 393. (a) The cell lysates were analyzed by western blotting with an anti–α-toxoid monoclonal antibody, and the results showed that pPG-α/*L. casei* 393, but not pPG/*L. casei* 393 and *L. casei* 393 expressed this toxoid. (b) Determination of the cell surface expression of the α-toxoid by laser confocal microscopy. The results revealed that there was clear-cut green fluorescence on the cell surface of pPG-α/*L. casei* 393, but not on pPG/*L. casei* 393. (c) The cell surface expression of the α-toxoid protein as analyzed by flow cytometry.

To confirm expression of the α-toxoid by the pPG-α/*L. casei* 393 constructed in this study, cell lysates of pPG-α/*L. casei* 393 were analyzed by a western blot assay. As shown in Figure 2(a), there was a specific protein on the immunoblot from pPG-α/*L. casei* 393 but not from pPG/*L. casei* 393 or *L. casei* 393, indicating that the α-toxoid protein was expressed by the genetically engineered pPG-α/*L. casei* 393 and was recognized by the anti–α-toxoid monoclonal antibody. Moreover, expression vector pPG-T7g10 is a cell surface expression system. Therefore, to analyze cell surface expression of the α-toxoid protein, laser confocal microscopy and flow cytometry assays were performed, and the results suggested that there were clear-cut green fluorescent signals on the cell surface of pPG-α/*L. casei* 393 observed by laser confocal microscopy, but not on pPG/*L. casei* 393 (Figure 2(b)). In addition, the number of pPG-α/*L. casei* 393 cells with a fluorescent signal relative to the total number of bacterial cells was significantly higher than that of pPG/*L. casei* 393 as analyzed by flow cytometry (Figure 2(c)), suggesting that the α-toxoid protein was expressed and displayed on the cell surface of engineered strain pPG-α/*L. casei* 393.

### *Immune responses induced by pPG-α/*L. casei *393 in mice*

**Figure 3.**
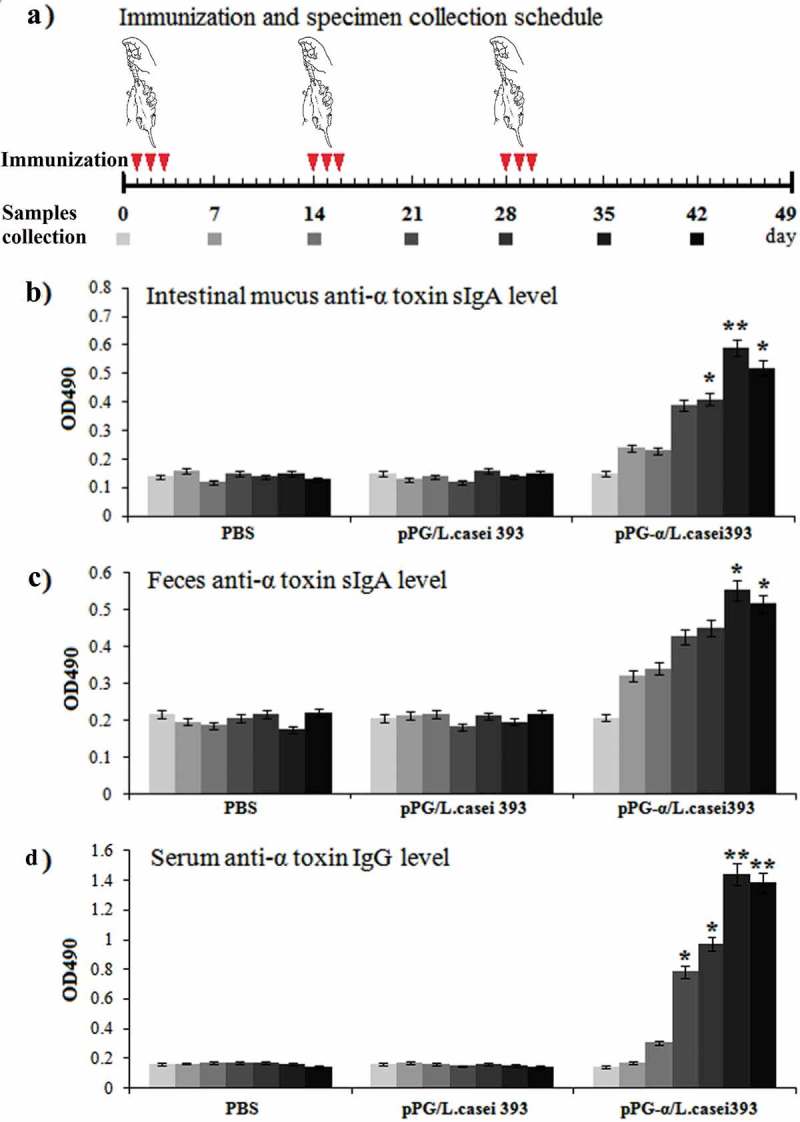
Determination of antigen-specific sIgA and IgG antibody levels in mice immunized with pPG-α/*L. casei* 393. (a) The immunization scheme, and the schedule of feces, serum, and intestinal mucus sampling. After immunization, immune samples were collected from the mice in each group on days 0, 7, 14, 21, 28, 35, and 42, and anti–α-toxoid sIgA levels in the intestinal mucus (b) and feces (c) and anti–α-toxoid IgG levels in serum (d) were detected by an ELISA. Bars represent the mean ± standard error in each group (*p < 0.05, **p < 0.01 as compared with groups “pPG/*L. casei* 393” and “PBS”).

**Figure 4.**
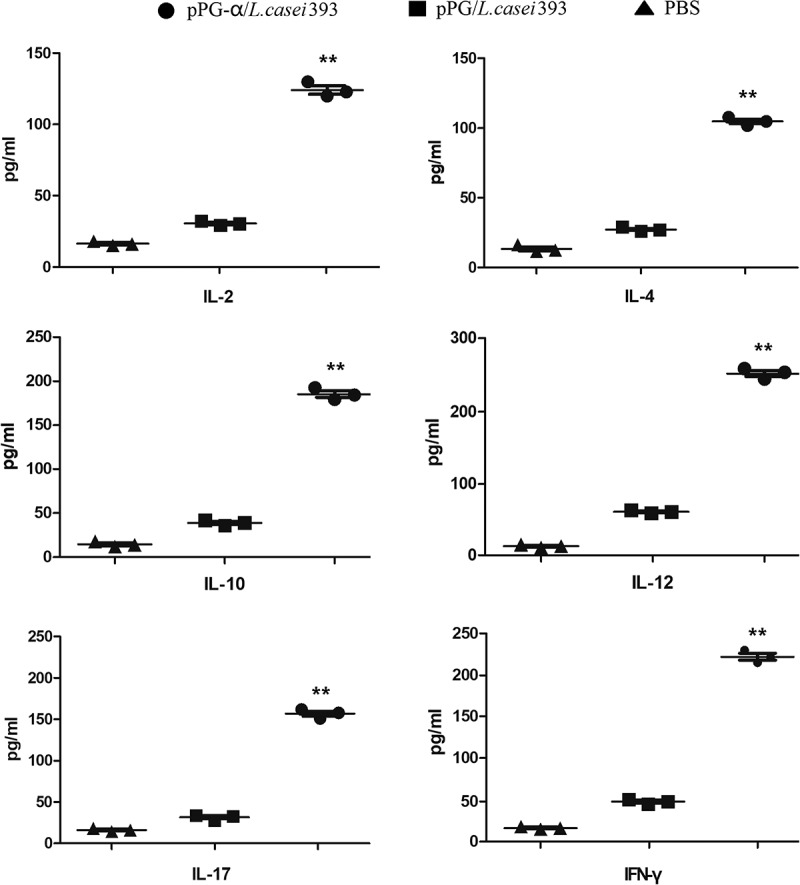
Determination of cytokine levels in the serum obtained from pPG-α/*L. casei* 393–immunized mice. The levels of cytokines IL-2, IL-4, IL-10，IL-12, IL-17, and IFN-γ in serum samples collected on day 35 post-immunization were determined by means of an ELISA kit. Results are mean ± SD (**p < 0.01 as compared with groups pPG/*L. casei* 393 and PBS).

The immunogenicity of engineered strain pPG-α/*L. casei* 393 in mice after oral immunization was evaluated by ELISAs, with special attention to the presence of a mucosal sIgA antibody and a serum IgG antibody. The scheme of immunization and sample collection is depicted in Figure 3(a). There were no substantial differences (*p *> 0.05) in sIgA and IgG antibody levels among the groups before immunization. After a second booster immunization, anti–α-toxoid specific mucosal sIgA antibody levels in the intestinal mucus (Figure 3(b)) and feces (Figure 3(c)), and specific anti–α-toxoid serum IgG antibody levels (Figure 3(d)) of mice in the pPG-α/*L. casei* 393 group were significantly higher (*p* ˂ 0.05) as compared to groups “pPG/*L. casei* 393” and “PBS.” Nonetheless, no significant changes in anti–α-toxoid sIgA or IgG levels were observed in mice that received pPG/*L. casei* 393 or PBS (Figure 3(b–c)). Therefore, the above results indicated that immunization with pPG-α/*L. casei* 393 induced specific mucosal and systemic immune responses. The levels of cytokines IL-2, IL-4, IL-10, IL-12, IL-17, and IFN-γ in the serum samples collected on day 35 post-immunization were also determined, and significantly higher cytokine levels (*p* ˂ 0.01) were observed in mice orally immunized with pPG-α/*L. casei* 393 as compared to group pPG/*L. casei* 393 or PBS (Figure 4). In addition, it is worth noting that the concentrations of these cytokines in the serum of mice in the pPG/*L. casei* 393 group were higher than those in the PBS group, suggesting that the nonengineered probiotic itself can directly enhance nonspecific immunity of the body.

### *The neutralization ability of the sIgA and IgG induced by pPG-α/*L. casei *393 toward the α-toxin*

**Figure 5.**
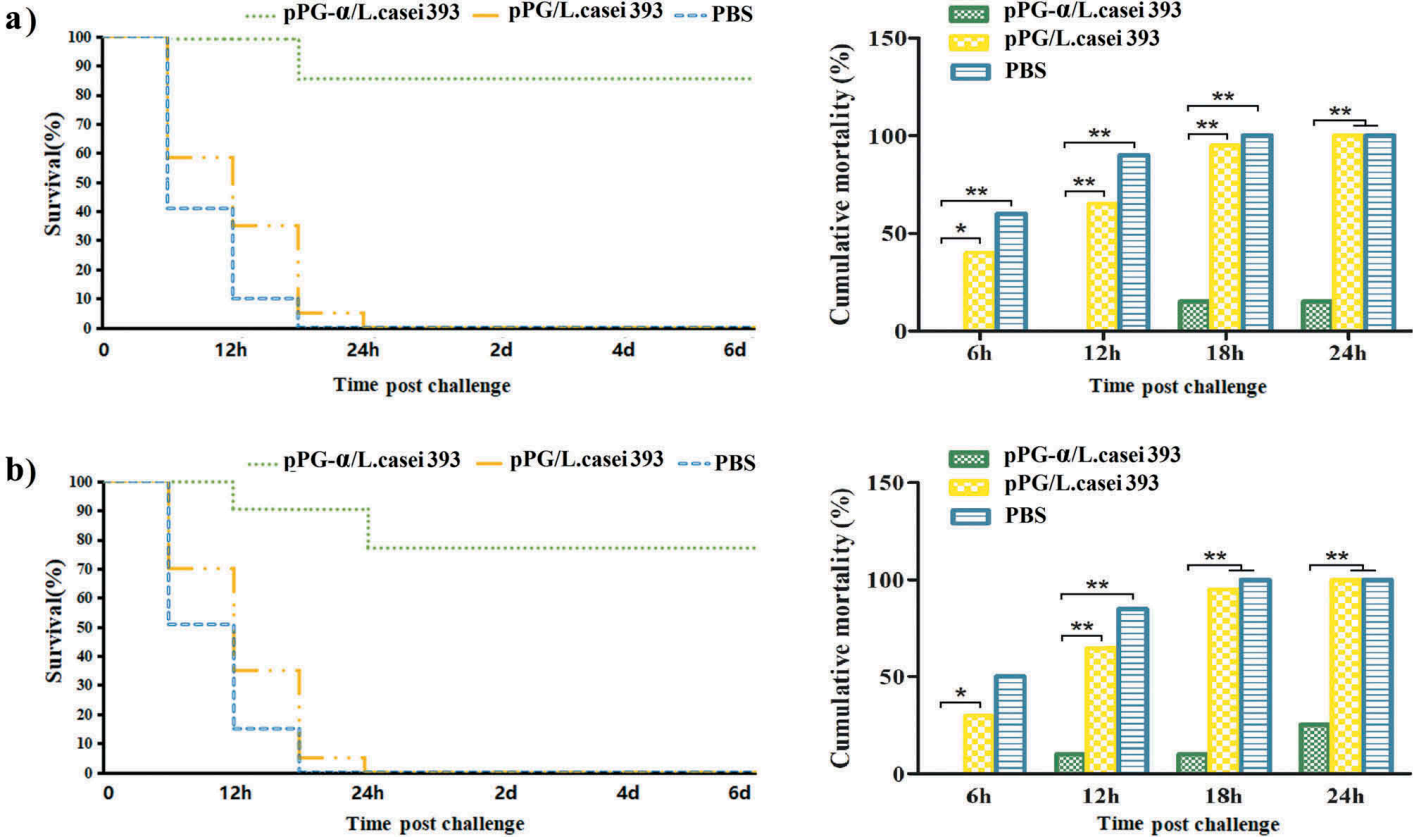
Neutralizing abilities of antibodies induced in immunized mice against *C. perfringens* natural α-toxin. *C. perfringens* natural α-toxin was treated with the serum IgG and mucosal sIgA antibodies obtained from the immunized mice in each group, followed by a challenge to the mice to evaluate the neutralizing abilities of the antibodies. A high survival rate was registered among the mice challenged with the α-toxin treated with IgG (a) or sIgA antibodies (b) induced by pPG-α/*L. casei* 393, whereas mice challenged with the α-toxin treated with antibodies obtained from mice immunized with pPG/*L. casei* 393 and PBS all died.

In this experiment, mice were challenged with *C. perfringens* natural α-toxin that was treated with the serum IgG or mucosal sIgA antibody isolated from the immunized mice in each group to evaluate the neutralizing abilities of the IgG or sIgA antibodies. The results showed that the serum IgG and mucosal sIgA antibodies induced by pPG-α/*L. casei* 393 had an effective neutralizing ability toward *C. perfringens* natural α-toxin, thereby resulting in a high survival rate in mice challenged with the α-toxin treated with IgG (85%; Figure 5(a)) and α-toxin treated with sIgA (75%; Figure 5(b)). At the same time, the cumulative mortality rate in mice of the probiotic vaccine group was significantly lower (*p *< 0.01) than that among mice of the pPG/*L. casei* 393 group or PBS group at each time point. In contrast, mice challenged with *C. perfringens* natural α-toxin – treated with antibodies obtained from mice orally immunized with pPG/*L. casei* 393 or PBS – all died within 24 h.

### Determination of lymphocyte proliferation

**Figure 6.**
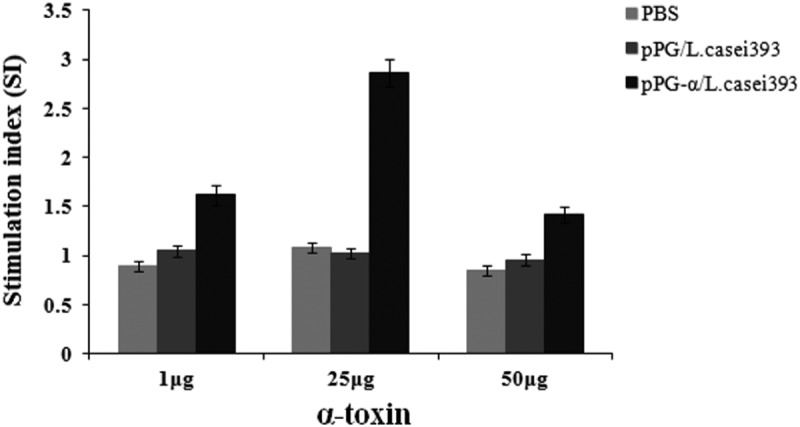
Lymphoproliferation. Splenocytes of the mice in each group were prepared on day 42 after primary immunization and restimulated with the purified α-toxoid protein. Next, lymphoproliferation was detected by the MTT assay. Results were expressed as a stimulation index. Bars represent the mean ± standard error in each group.

On day 42 after primary immunization, splenocytes from mice in each group were isolated and restimulated with the purified α-toxoid protein expressed by strain pET-32a-α/BL21, followed by proliferation evaluation in the MTT assay. As a result, significant splenocyte proliferation was observed in the group of mice orally immunized with pPG-α/*L. casei* 393 but not in the group of mice that received pPG/*L. casei* 393 or PBS (Figure 6). Namely, a significant change was noted in the vaccine group stimulated with the purified α-toxoid protein at 25 μg/mL.

### *Efficiency of the probiotic vaccine protection against* C. perfringens *α-toxin*

**Figure 7.**
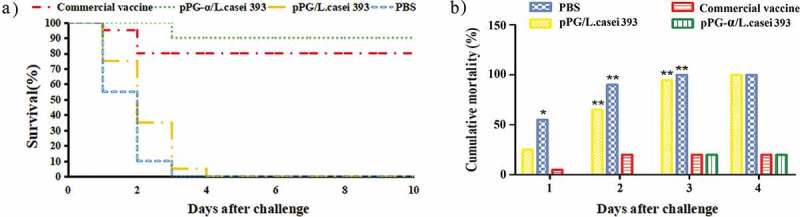
Protection efficiency against a *C. perfringens* natural α-toxin challenge in mice orally immunized with recombinant pPG-α/*L. casei* 393.

**Figure 8.**
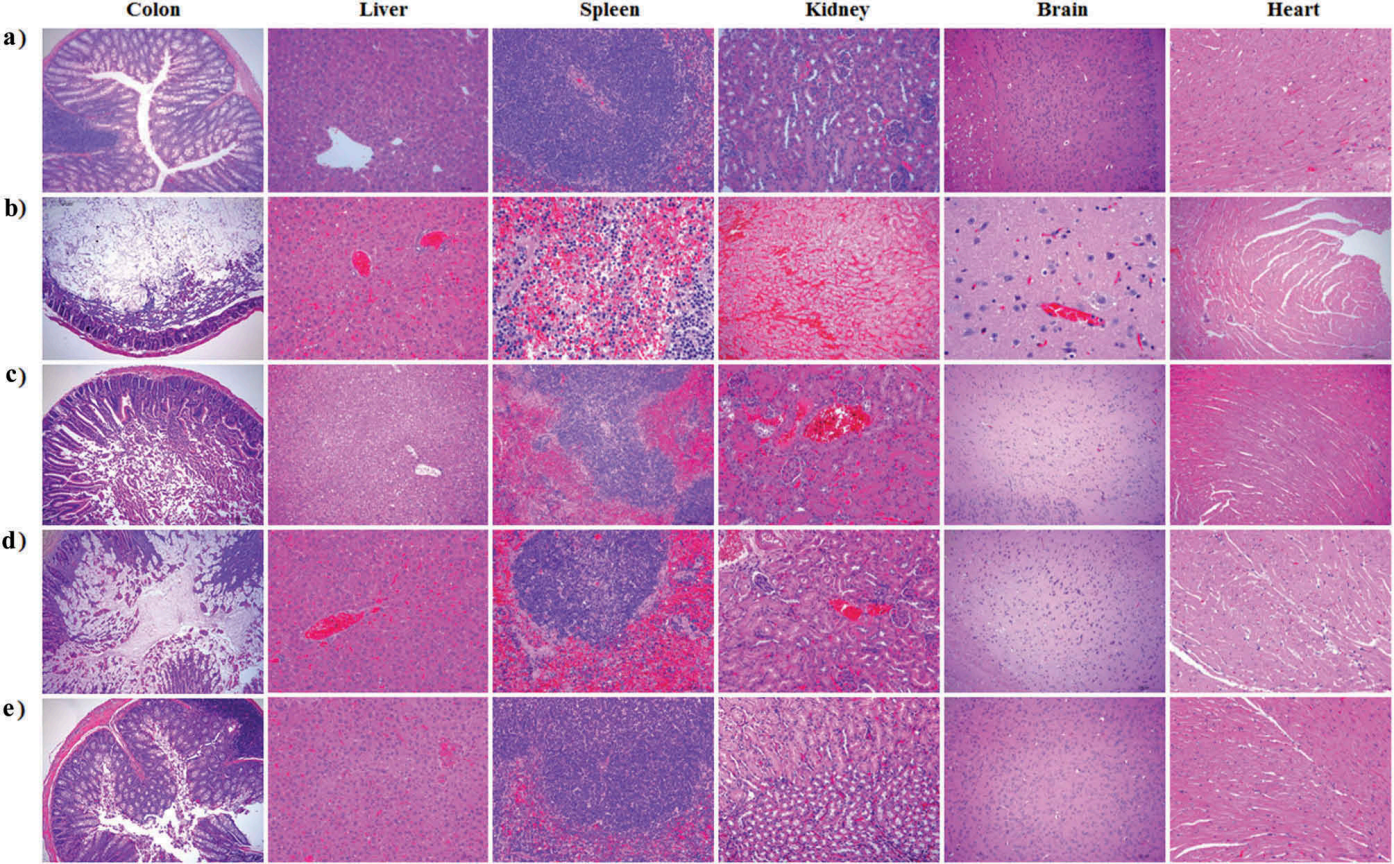
Histopathological examination of immunized mice after a challenge with the α-toxin. No histopathological changes were observed in the heart, liver, spleen, kidneys, colon, and brain of mice in the normal control group (a). Histopathological changes after the challenge were noticed in the tissues of mice orally vaccinated with PBS (b) or pPG/*L. casei* 393 (c). In the commercial vaccine group (d), histopathological changes were observed in the liver, kidneys, and colon of the mice, particularly in the colon after the challenge, whereas little or no histopathological changes were observed in the tissues of mice orally immunized with pPG-α/*L. casei* 393 (e). Tissue sections were stained with hematoxylin and eosin and photographed at 40× magnification.

In this study, a challenge test was performed with the natural α-toxin combined with *C. perfringens* toxinotype A to evaluate the protection efficiency of engineered strain pPG-α/*L. casei* 393 in mice on day 35 post-immunization. After the challenge with 1 × LD_100_ of *C. perfringens* natural α-toxin combined with 200 μL of *C. perfringens* type A (10^9^ CFU/mL), the survival rates of mice in the probiotic vaccine (pPG-α/*L. casei* 393) group and in the commercially available inactivated *C. perfringens* toxinotype A vaccine group were 90% and 80%, respectively, whereas the mice in the pPG/*L. casei* 393 group and PBS group all died (Figure 7). These data suggested that effective protection can be provided by pPG-α/*L. casei* 393 via oral immunization. Moreover, after the challenge, histopathological changes in the heart, liver, spleen, kidneys, brain, and intestines of mice in each group were examined. There were no pathological changes in the normal control group (Figure 8(a)). The mice in the PBS group (Figure 8(b)) and pPG/*L. casei* 393 group (Figure 8(c)) developed severe symptoms and obvious histopathological damage, including colonic mucosal epithelial necrosis and severe shedding, intestinal villus shortening, disruption of structural integrity and disintegration of erythrocytes in the red pulp of the spleen, hemoglobin precipitation, and macrophage infiltration. In the commercial vaccine group (Figure 8(d)), colonic-mucosal-epithelium necrosis, celluloselike exudation in the gut, intestinal villus shedding, liver congestion, and slight congestion of the kidneys were noted (Figure 8(d)). In contrast, mice in the pPG-α/*L. casei* 393 group had slight congestion of the intestinal mucosa, intestinal villus shedding, mild congestion in the liver and kidneys, and no abnormal histopathological changes in the heart, spleen, or brain (Figure 8(e)).

## Discussion

The main virulence agents of *C. perfringens* – as an opportunistic pathogen in the gastrointestinal tract of livestock and poultry – are exotoxins, and the α-toxin is currently one of the most important exotoxins that can be absorbed by the intestinal mucosa and cause human and animal diseases [9,28]. Therefore, an immunological method based on induction of effective intestinal mucosal immunity against exotoxins is a promising approach to prevention of the relevant diseases. Oral immunization is a desirable method for inducing both local and systemic immune responses against gastrointestinal infection by a pathogen [24]. In recent years, LAB have been widely used to develop oral vaccines as delivery vehicles, particularly species of the genus *Lactobacillus*, which have low innate immunogenicity and manifest strong adhesion to the intestinal mucosa; they have shown a promising potential as mucosal vaccine vectors with the capacity for delivering an antigen and eliciting both mucosal and systemic antigen-specific responses [24,26,27]. In the present study, to develop an oral probiotic vaccine against *C. perfringens* α-toxin, a genetically engineered *Lactobacillus* strain, pPG-α/*L. casei* 393, expressing the α-toxoid protein was constructed, and its immunogenicity and immune protection against a *C. perfringens* α-toxin challenge were evaluated on BALB/c mice as an animal model.

In this work, *Lactobacillus* constitutive expression vector pPG-T7g10 constructed in our lab was employed to deliver the α-toxoid protein. The pPG-T7g10 vector contains a T7g10 enhancer derived from gene 10 of bacteriophage T7; this sequence can enhance translation and expression of proteins [29]. Moreover, the pPG-T7g10 vector contains a gene encoding the PgsA anchor protein, which is a transmembrane protein derived from *Bacillus subtilis*; the amino terminus of a target protein can be fused to the PgsA protein and be stabilized by anchoring on the surface of the cell to facilitate the target protein’s function [30,31]. In this study, a western blot showed that the protein of interest (α-toxoid protein) can be constitutively expressed by genetically engineered strain pPG-α/*L. casei* 393 and can be accurately recognized by an anti–α-toxoid monoclonal antibody. Furthermore, display of the α-toxoid protein on the cell surface of strain pPG-α/*L. casei* 393 was confirmed by laser confocal microscopy and flow cytometry. Therefore, the membrane-associated antigen-producing genetically engineered *Lactobacillus* strain as an oral vaccine may help the host to capture antigens and effectively elicit antigen-specific immune responses.

Oral vaccination can induce mucosal immune responses, especially an intestinal one, and an effective intestinal mucosal immune response requires production of both mucosal sIgA and serum IgG antibodies against the infectious agent, which have been proven as a valid approach against the colonization by pathogens and against their further spread to the systemic circulation [32,33]. *C. perfringens* α-toxin mainly has to be absorbed through the intestinal mucosa to cause a disease; therefore, the development of an oral vaccine that can effectively induce specific sIgA-based mucosal and IgG-based humoral immune responses is important for survival of the host after an α-toxin challenge. Mucosal sIgA is the predominant antibody on the mucosal surface and is produced locally in the amount that exceeds that of other immunoglobulins and constitutes the first line of defense for the host against infections [34,35]. In this study, BALB/c mice served as an animal model for evaluation of the immunogenicity of pPG-α/*L. casei* 393 in terms of induction of mucosal and systemic responses against the α-toxin. An antigen-specific sIgA antibody in the feces and intestinal mucus and an antigen-specific IgG antibody in serum were quantified after oral immunization. Our data revealed that significant amounts of a specific anti–α-toxoid mucosal sIgA antibody in the intestinal mucus and feces and of a specific serum IgG antibody were induced in the mice orally immunized with pPG/*L. casei* 393 after a second booster immunization. In contrast, there were no significant changes (*p *> 0.05) of sIgA and IgG antibody levels in the mice that received pPG/*L. casei* 393 or PBS. Moreover, mucosal sIgA and serum IgG antibodies induced by the probiotic vaccine turned out to have good abilities to neutralize *C. perfringens* natural α-toxin. Our results indicate that genetically engineered *Lactobacillus* strain pPG-α/*L. casei* 393 as an oral vaccine can effectively induce antigen-specific mucosal and systemic immune responses against *C. perfringens* α-toxin and has good immunogenicity.

Lymphocyte proliferation positively correlates with cellular immunity. In this study, proliferation of spleen lymphocytes from mice orally immunized with pPG-α/*L. casei* 393 was confirmed, suggesting that a cellular immune response can be induced by the probiotic vaccine. The balance or ratio of T helper 1 (Th1) and Th2 cells is important for a healthy immune response. One study suggests that *L. casei* can promote the process of innate immune responses and activate Th1-mediated immune responses [36]. IFN-γ produced by CD8^+^ T cells and certain CD4^+^ T cells (especially Th1 cells) can enhance the activity of Th1 cells and promote cellular immune function [37]. The Th2-type immune response is marked by secretion of cytokine IL-4 and induction of specific antibodies [38]. Antigen-specific B-cell responses to mucosally delivered proteins are dependent upon CD4^+^ T helper cells, and the frequency of Th1 and Th2 cell responses after oral immunization may determine the magnitude and isotype of mucosal antibody responses. In this study, we examined Th1-type (IFN-γ, IL-2, and IL-12) and Th2-type (IL-4 and IL-10) cytokine levels in the serum of mice immunized with pPG-α/*L. casei* 393 by ELISAs. Significantly higher amounts (*p *< 0.01) of these cytokines were found to be secreted into the serum of mice in the pPG-α/*L. casei* 393 group compared to group pPG/*L. casei* 393 or PBS. In addition, cytokine IL-17 was effectively induced by the oral probiotic vaccine; this mechanism may fight against pathogen invasion at different phases and locations of infection. Moreover, our results confirmed that a mixed Th1- and Th2-type immune response can be induced by the genetically engineered *Lactobacillus*.

An effective vaccine against *C. perfringens* should be capable of eliciting potent immune responses against exotoxins: the main virulence factors of *C. perfringens*. Therefore, to confirm the protection efficacy of pPG-α/*L. casei* 393 as an oral vaccine, a challenge test was performed on mice by means of the crude α-toxin of *C. perfringens* coupled with *C. perfringens* type A after the second booster immunization. A commercial inactivated *C. perfringens* type A vaccine served as a vaccine control. Our results revealed that the survival rates of mice in the probiotic vaccine group and in the commercial vaccine group reached 90% and 80%, respectively, whereas none of the mice in the pPG/*L. casei* 393 and PBS groups survived. This finding indicates that the mucosal and systemic immune responses induced by pPG-α/*L. casei* 393 via oral immunization can provide effective protection in mice to neutralize an attack by *C. perfringens* type A together with its crude α-toxin. Moreover, histopathological changes were detected in the liver, spleen, kidneys, and colon of mice in the pPG/*L. casei* 393 and PBS groups exposed to the α-toxin challenge, whereas no significant changes were observed in the tissues of mice from the probiotic vaccine group before and after the challenge. All these results confirm that the toxoid of *C. perfringens* α-toxin as an immunogen expressed by strain pPG-α/*L. casei* 393 induced effective protective immunity against the crude α-toxin challenge.

In summary, the genetically engineered strain pPG-α/*L. casei* 393 constructed in this study expressing the toxoid of *C. perfringens* α-toxin was able to effectively induce potent antigen-specific mucosal and humoral immune responses and to protect murine hosts from a *C. perfringens* natural α-toxin challenge, suggesting that the engineered strain is a promising candidate vaccine against the α-toxin.
